# Basic concepts of mixture toxicity and relevance for risk evaluation and regulation

**DOI:** 10.1007/s00204-023-03565-6

**Published:** 2023-08-24

**Authors:** Denise Bloch, Patrick Diel, Bernd Epe, Michael Hellwig, Alfonso Lampen, Angela Mally, Doris Marko, María A. Villar Fernández, Sabine Guth, Angelika Roth, Rosemarie Marchan, Ahmed Ghallab, Cristina Cadenas, Patrick Nell, Nachiket Vartak, Christoph van Thriel, Andreas Luch, Sebastian Schmeisser, Matthias Herzler, Robert Landsiedel, Marcel Leist, Philip Marx-Stoelting, Tewes Tralau, Jan G. Hengstler

**Affiliations:** 1 Department of Pesticides Safety, German Federal Institute for Risk Assessment (BfR), Berlin, Germany; 2 Department of Molecular and Cellular Sports Medicine, Institute of Cardiovascular Research and Sports Medicine, German Sport University Cologne, Cologne, Germany; 3 Institute of Pharmaceutical and Biomedical Sciences, University of Mainz, Mainz, Germany; 4 Chair of Special Food Chemistry, Technical University Dresden, Dresden, Germany; 5 Risk Assessment Strategies, German Federal Institute for Risk Assessment (BfR), Berlin, Germany; 6 Department of Toxicology, University of Würzburg, Würzburg, Germany; 7 Department of Food Chemistry and Toxicology, Faculty of Chemistry, University of Vienna, Vienna, Austria; 8 Department of Toxicology, Leibniz Research Centre for Working Environment and Human Factors (IfADo), Dortmund, Germany; 9 Department of Chemical and Product Safety, German Federal Institute for Risk Assessment (BfR), Berlin, Germany; 10 Department of Experimental Toxicology and Ecology, BASF SE, Ludwigshafen, Germany; 11 Pharmacy, Pharmacology and Toxicology, Free University of Berlin, Berlin, Germany; 12 Department of In Vitro Toxicology and Biomedicine, Inaugurated by the Doerenkamp-Zbinden Foundation, University of Konstanz, Constance, Germany; 13 Department of Forensic Medicine and Toxicology, Faculty of Veterinary Medicine, South Valley University, Qena, 83523 Egypt

## Abstract

Exposure to multiple substances is a challenge for risk evaluation. Currently, there is an ongoing debate if generic “mixture assessment/allocation factors” (MAF) should be introduced to increase public health protection. Here, we explore concepts of mixture toxicity and the potential influence of mixture regulation concepts for human health protection. Based on this analysis, we provide recommendations for research and risk assessment. One of the concepts of mixture toxicity is additivity. Substances may act additively by affecting the same molecular mechanism within a common target cell, for example, dioxin-like substances. In a second concept, an “enhancer substance” may act by increasing the target site concentration and aggravating the adverse effect of a “driver substance”. For both concepts, adequate risk management of individual substances can reliably prevent adverse effects to humans. Furthermore, we discuss the hypothesis that the large number of substances to which humans are exposed at very low and individually safe doses may interact to cause adverse effects. This commentary identifies knowledge gaps, such as the lack of a comprehensive overview of substances regulated under different silos, including food, environmentally and occupationally relevant substances, the absence of reliable human exposure data and the missing accessibility of ratios of current human exposure to threshold values, which are considered safe for individual substances. Moreover, a comprehensive overview of the molecular mechanisms and most susceptible target cells is required. We conclude that, currently, there is no scientific evidence supporting the need for a generic MAF. Rather, we recommend taking more specific measures, which focus on compounds with relatively small ratios between human exposure and doses, at which adverse effects can be expected.

## Introduction

Humans are exposed to large numbers of substances, including those that are man made, either intentionally or unintentionally formed, as well as naturally occurring compounds. These substances are present in our food, environment and workplaces (Huhn et al. 2021). While risk assessment is well established for single compounds, simultaneous exposure to multiple substances is a challenge for risk evaluation and basic toxicological research. The toxicity elicited by exposure to multiple substances is referred to as “mixture toxicity” (Sarigiannis and Hansen 2012; Shaw 2014). It is important to understand how different substances act and interact when concomitantly present in the human body and whether such interactions influence toxic effects. Together, they may lead to additive or even more than additive (synergistic) effects as compared to the toxicity of the individual components (Delfosse et al. 2021; Elcombe et al. 2022).

Recently, concern that mixtures of substances could cause unknown or elevated known toxicity in humans led to a discussion on how to account for potential combined effects of mixture components (Tralau et al. 2021). A generic “Mixture Assessment/Allocation Factor” (MAF) (KEMI 2021) was proposed as a risk management measure by Swedish (KEMI) and Dutch (RIVM) EU Member State authorities and taken up by the European Commission in the “EU Chemicals Strategy for Sustainability” (European Commission 2020). A generic MAF reduces the acceptable exposure limit (AEL) by a factor of, for example, 2, 5 or 10. The AEL is usually determined as the risk characterisation ratio (RCR)/risk quotient (RQ), *i.e.* the quotient of the exposure of an individual and a health-based guidance value (HBGV) such as the acceptable/tolerable daily intake (ADI/TDI) or the derived no-effect level (DNEL) representing the highest dose not assumed to cause adverse effects in humans. In contrast to a specific data-driven approach, a generic MAF is equally applied to all substances to which humans are exposed, regardless of their individual potential to contribute to mixture effects. However, before implementing untargeted and universal measures, scientific evidence, mechanistic plausibility and the uncertainties of mixture toxicity should be explored.

In the current debate on toxic effects of mixtures, it is sometimes difficult to differentiate between well-established concepts and hypotheses that have not yet been proven since the available experimental data is inconclusive. To facilitate the discussion, we first describe established mechanisms of substance interactions. For the sake of better recognition, we named these concepts “multi-headed dragon” and “synergy of evil”. In the “multi-headed dragon” concept, several substances—symbolised by the heads of the dragon—act by the same mechanism or mechanisms converging in the same molecular key event, leading to effects in the same biological target, e.g. in a certain cell type. In the “synergy of evil” concept, one substance enhances the toxic effect of another. This can occur because of the inhibition of enzymes involved in metabolic detoxification or transporters responsible for the excretion of substances resulting in increased substance concentrations (toxicokinetic “synergy of evil”). Moreover, different mechanisms (with no overlap in their key events) may indirectly enhance each other (toxicodynamic “synergy of evil”). We also look at the prevalent assumption that large numbers of substances to which human populations are exposed at very low, individually harmless doses compound to cause adverse effects (Dinca et al. 2023). We named this assumption the “revolting dwarfs” hypothesis. Based on the available data we conclude that mixture toxicity in mammals and humans, if applicable, largely follows the “multi-headed dragon” concept and discuss conditions for grouping substances that may act additively when present at relevant exposure concentrations over a relevant exposure duration and a critical time window. We further conclude that there is currently neither experimental evidence nor a plausible mechanism supporting the “revolting dwarfs” hypothesis. Consequently, there is also no need for generic protective approaches against health impacts from multi-chemical exposures at very low levels. Instead, we highlight the need to further develop and refine concepts for targeted mixture toxicity assessment.

## Thresholds of toxic effects and concepts of risk evaluation of individual substances

A basic assumption in toxicology is that adverse effects of substances are dose dependent. Lower doses of a substance will cause lower or no toxicity in a given organism compared to higher doses. Without this, life could not exist as it constantly faces chemical challenges, be it from substances of natural or synthetic origin. Unsurprisingly, all organisms, therefore, dedicate significant parts of their biochemistry to the metabolism, excretion and inactivation of harmful substances, which emanate from both endogenous and exogenous sources (Monosson 2012). Particularly regarding food it should be noted that our daily diets routinely consist of complex mixtures that contain a plethora of substances, for which efficient metabolic detoxification is known. Without such highly efficient mechanisms that cover a vast chemical space, many substances would show a much higher degree of toxicity. Based on our mechanistic understanding, adverse effects will not manifest below a specific dose. The molecular basis for this assumption is that the interaction of a toxic agent with its biological target, which is responsible for the effect, is negligible below a certain concentration or—for irreversible interactions—below a certain cumulative concentration. An exception are carcinogens with a direct genotoxic mode of action where the dose at which no carcinogenicity occurs may be very low and difficult to identify (Bolt et al. 2004; Hengstler et al. 2003; Nohmi 2018). It should be noted, however, that nowadays there is increasing discussion that agents acting via genotoxic and mutagenic mechanisms may also exhibit non-linear dose–response relationships that allow a threshold to be established (Guth et al. 2023; Hartwig et al. 2020). Nevertheless, there is general agreement that for all other adverse effects—at least theoretically—an identifiable exposure threshold exists below which no adverse effects occur.

**Fig. 1:**
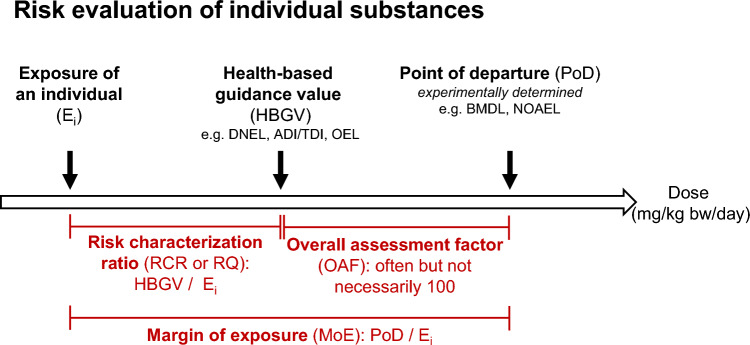
Principle of risk evaluation of individual substances. DNEL, derived no-effect level; ADI, acceptable daily intake; TDI, tolerable daily intake; OEL, occupational exposure limit; BMDL, benchmark dose lower confidence limit; NOAEL, no observed adverse effect level

In dose-dependent animal experiments, usually two values are determined as the so-called “points of departure” (PoDs) for risk assessment: the no observed adverse effect level (NOAEL), the highest dose that has not caused adverse health effects in that experiment, and the lowest observed adverse effect level (LOAEL), the lowest dose at which a measurable adverse effect in vivo has been observed. Both parameters are usually expressed as mg per kg body weight per day (mg/kg bw/day) for exposures via the oral route (Fig. 1). For most substances, the NOAEL and LOAEL were derived from sub-chronic or chronic studies with rodents. Upon data availability, NOAELs and LOAELs may also be derived from human epidemiological or clinical studies.

Benchmark dose (BMD) modelling has been implemented into regulatory toxicology to provide a more robust and objective means of threshold derivation. It considers the whole dose–response curve while referring to a defined critical effect size and accounting for uncertainty in the derived PoD. Usually, this is reflected by using the BMD lower confidence limit (BMDL) rather than the BMD itself. On average, the BMD- and NOAEL-based approaches will in general result in comparable PoDs, although substantial differences, *e.g.* within one order of magnitude, are possible in individual cases (EFSA Scientific Committee et al. 2022). Consequently, already derived NOAELs can remain in place, but should be reviewed upon substance re-evaluation using the BMD approach as recommended by EFSA.

In risk assessment, PoDs like BMDL or NOAEL enable decisions on authorisation and safe use levels. However, both exposure and threshold values are estimated with uncertainties. The observed PODs clearly depend on the sensitivities of the measurements carried out and there is no guarantee that all relevant health effects of the compounds are covered by the tests applied. Hence, human exposure should be a rather small fraction of the PoD. There are different approaches to determine health-based guidance values (HBGVs), representing the maximum exposure of humans to a substance that is not expected to result in an appreciable health risk, taking into account the uncertainties of the safety data and the exposure conditions. Examples of HBGVs are the DNEL, ADI/TDI and Occupational Exposure Limits (European Commission 2018). To derive HBGVs the PoD is usually divided by an overall assessment factor (OAF) combining a set of individual assessment factors (AFs) accounting for, e.g. inter- or human intraspecies variability (Dourson et al. 1996; EFSA Scientific Committee 2012; Lehman and Fitzhugh 1954; World Health Organization 2009; World Health Organization et al. 1987). Historically, an OAF of 100 has been routinely applied in his context, but deviations are possible (both in the direction of higher and lower values), *e.g.* depending on the species used in the animal experiment or if substance-specific AF were available. In more recent times, this concept has been further extended, *inter alia* by implementing the concept of Allometric Scaling under Regulation (EC) 1907/2006 [REACH Regulation (European Commission 2006)] or by the representation of AFs as distributions rather than point estimates (Chiu and Slob 2015; World Health Organization and International Programme on Chemical Safety 2018) to enable probabilistic hazard and risk characterisation.

## Concepts of how several substances in mixtures can interact to cause adverse effects

Chiefly, there are two concepts of concern that explain how substances in a mixture can interact to increase adverse effects beyond what might be caused by their individual components. In the present work, we further explore these concepts under the names of “multi-headed dragon” and “synergy of evil”.

### Concept of the “multi-headed dragon”

**Fig. 2:**
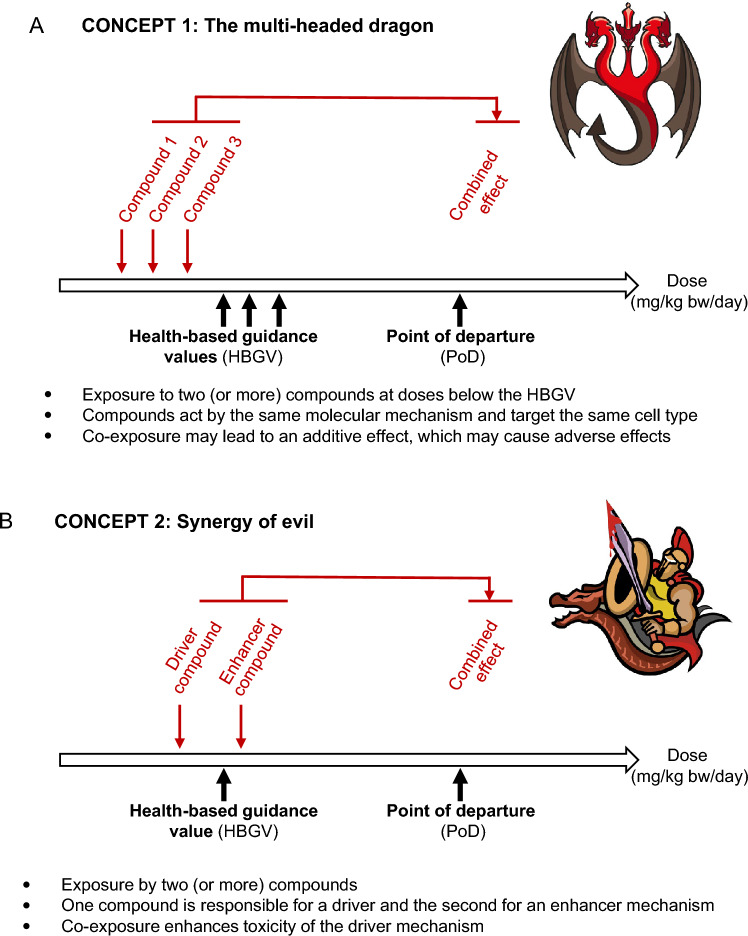
Concepts of mixture toxicity. **A** The Multi-Headed Dragon. **B** The Synergy of Evil

Substances in a mixture may exhibit additive effects of toxicity if they act by the same molecular mechanism in identical target cells, even if they are present below their respective HBGVs and, thus, are considered safe individually (Fig. 2A). We dub this the concept the “multi-headed dragon”: the mechanism that results in injury—here symbolised by the dragon head—is multiplied to create an even more dangerous dragon. In toxicology, this principle is well documented, *e.g.* for polychlorinated dibenzo-p-dioxins (PCDDs), polychlorinated dibenzofurans (PCDFs), and polychlorinated biphenyls (PCBs), all of which cause adverse effects predominantly by the activation of the aryl hydrocarbon receptor (AhR) (Beischlag et al. 2008; Bradshaw and Bell 2009; Fernandez-Salguero et al. 1996; Gonzalez and Fernandez-Salguero 1998; Peters and Gonzalez 2011; Peters et al. 1999). For these substances, mixture effects can be assessed using toxic equivalents (TEQs), which represent the toxicity of a substance as a fraction of the most toxic substance of this type (Safe 1998). Each compound is allotted a toxic equivalency factor (TEF), rating its toxicity in relation to the most toxic congener (e.g. 2,3,7,8-TCDD), which is assigned the highest TEF of 1.0. To evaluate the mixture effects of such compounds, concentrations of the individual substances (*C*_i_) are multiplied by their individual TEFs (*C*_i_ × TEF) and the sum of all *C*_i_ × TEF is calculated. It should be noted that dioxin-like substances may also act by AhR-independent mechanisms (Neumann 1996; Peters and Gonzalez 2011; Safe 1997). However, according to the concept of the multi-headed dragon, which requires activity via the same mechanism, they are of minor relevance for mixture effects within this compound class. Moreover, some PCDDs, PCDFs and PCBs may act less than additively, since toxicokinetics as well as bioavailability are not considered in this risk assessment approach (Peters and Gonzalez 2011; Safe 1997).

The TEF approach has also been discussed for the assessment of mixture toxicity for compound groups other than dioxin-like compounds, such as perfluoroalkyl substances, although this approach is still challenging (Peters and Gonzalez 2011; Scialli et al. 2007). There are two classes of perfluoroalkyl substances: perfluorinated carboxylic acids (PFCAs) and perfluorosulfonic acids (PFSAs), which are suspected to cause hepatotoxicity, developmental toxicity, immunotoxicity and tumorigenesis (Colnot and Dekant 2021; Kudo and Kawashima 2003; Lau et al. 2007, 2004). However, in contrast to dioxin-like substances that cause adverse effects predominantly via a single receptor, the AhR, PFCAs and PFSAs may act via numerous nuclear receptors, including PPARα, PPARγ, PPARβ/δ, CAR, PXR, liver X receptor α and ERα (Fenton et al. 2021; Peters and Gonzalez 2011). This leads to a complex scenario because it is insufficiently clear via which receptors individual substances cause adverse effects or how these receptors may interact with each other quantitatively. In addition, the individual PFCAs and PFSAs exhibit toxicokinetic differences, a varying degree of interspecies variability and target different tissues (Peters and Gonzalez 2011). Therefore, the development of a TEF concept appears to be difficult for perfluoroalkyl substances.

A precondition for setting up a TEF concept for perfluoroalkyl substances would be to establish a database containing information on the degree to which individual perfluoroalkyl substances activate the aforementioned receptors and the tissues affected. This concept may be extended to other substances, such as triazole fungicides. It has been shown recently that two of these fungicides, propiconazole and tebuconazole, act in a concentration-additive manner on the RNA expression of drug metabolism-related genes, such as CYP3A4 (Knebel et al. 2018). However, combined exposure to just these two substances is complex since some effects may be additive, such as CYP3A expression, and some antagonistic, such as CAR activation (Knebel et al. 2018). These findings suggest that the probability of additive effects of substances in a mixture will decrease with the number of different molecular targets they act upon and with the number of different cell types and tissues that are affected due to differences in toxicokinetics (EFSA Scientific Committee et al. 2021). In addition, the exposure to the mixture components needs to be relatively constant, spanning at least a critical time window to be considered relevant for potential mixture effects.

For life-long exposure, more than additive effects have been reported in some cases (Dinca et al. 2023). Confirmations and mechanistic details of such studies require further research. Based on the understanding that different chemicals may affect similar processes in biology, EFSA has developed the concept of cumulative assessment groups (CAG). An example would be pesticides that “affect the motor division of the nervous system” (van Klaveren et al. 2021). Many pesticides in this CAG inhibit acetylcholine esterase. Thus, they affect the same molecular target and signalling pathway. However, there are also entirely different targets, such as sodium channels, on other cells or parts of the body that affect motor behaviour. This exemplifies that the original CAG concept is not molecularly defined, but rather takes biological functions or pathological observations as starting points. The latter point is exemplified by the CAG for hepatotoxicants, which comprise, for instance, the group of compounds triggering hypertrophy or steatosis (Colnot et al. 2020). Another example is the CAG that refers to craniofacial malformations or neural tube defects (European Food Safety Authority et al. 2022). Many different targets and cell types may be involved in these pathologies. As the CAG concept is developed further, it will include more pathologies (*e.g.* renal toxicity) and more molecular subdivisions (European Food Safety Authority et al. 2019). It can be expected that CAGs may be linked to adverse outcome pathways (AOP) or target pathways, when sufficient information is available on the relationship of pathological endpoints and the responsible target pathways. The examples of steatosis or neural tube defects show that many molecular targets and signalling pathways may converge on one given pathology. This implies that also compounds affecting different parts of such a network of pathways may, in theory, act additively (or synergistically) with respect to the final adverse outcome.

### Concept of the “synergy of evil”

*Toxicokinetic “synergy of evil”.* The most frequently observed type of synergism is the toxicokinetic “synergy of evil”, where the toxic effect of one substance or a mixture of substances, named “driver(s)”, may be enhanced by a second substance (or mixture), named “enhancer” (Fig. 2B). For example, synergism is observed when the enhancer reduces the metabolic inactivation or excretion of the driver leading to a situation where more driver substance is present at the site of effect. Consequently, the toxicity induced by the driver will be increased by the enhancer, if the dose of the driver as a single substance is already above that of the LOAEL. If the dose of the driver (as a single substance) is below the HBGV, as shown in Fig. 2B, the addition of the enhancer can, in principle, increase the concentrations of the driver substance in an organism such that effect thresholds are exceeded. A further scenario causing “synergy of evil” is given when an enhancer substance induces enzymes that are responsible for metabolic activation of a driver substance.

**Fig. 3:**
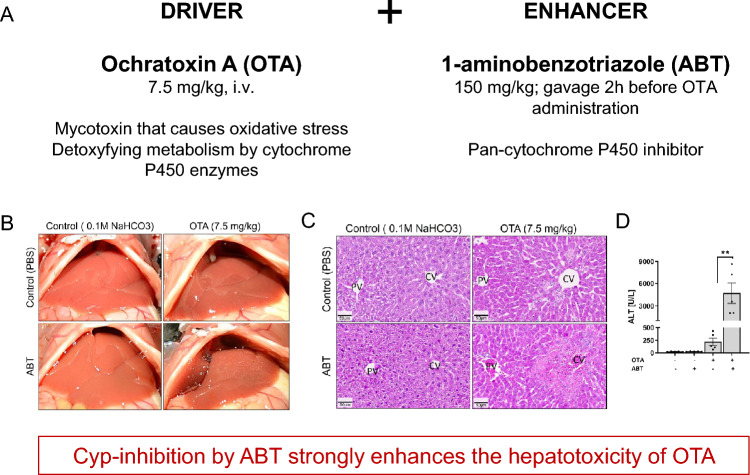
Example of Synergy of Evil of two substances of an experiment in mice (from Hassan et al. 2022a, b). **A** Doses of the driver (ochratoxin A) and enhancer (1-aminobenzotriazole) substance; **B** Gross pathology of the exposed mouse livers; **C** Histological analysis; **D** Serum activities of the liver enzyme alanine transaminase (ALT)

A recently published example of a toxicokinetic “synergy of evil” is the combined exposure of the mycotoxin ochratoxin A (OTA) with the unspecific pan-cytochrome P450 (CYP) inhibitor, 1-aminobenzotriazole (ABT) (de Montellano 2018) (Fig. 3A–D). One of the main mechanisms of action of OTA is its induction of oxidative stress (Ghallab et al. 2021; Hassan et al. 2022a; Kőszegi and Poór 2016; Mally and Dekant 2009; Ringot et al. 2006; Tao et al. 2018). Moreover, OTA is known to be detoxified by CYP enzymes (Hassan et al. 2022a). Under the conditions of the study, OTA alone induced only a slight increase in serum alanine transaminase (ALT) activity, a marker of hepatotoxicity. Histological analysis showed signs of weak hepatotoxicity as evidenced by the necrosis of individual hepatocytes around the central vein. In contrast, ABT alone had no effect on hepatotoxicity at the dose applied by Hassan et al. (2022b). However, combined exposure to OTA and ABT caused a hepatotoxic effect several orders of magnitude stronger than OTA alone, as evidenced by a massive increase in serum ALT and aspartate aminotransferase (AST) activities, as well as necrosis of the entire pericentral zone of the liver lobules (Fig. 3B–D). This example illustrates an important feature of the “synergy of evil”: synergism occurs between two different mechanisms. In the case of toxicokinetic “synergy of evil”, one of the two substances acts as a driver (here, OTA by inducing oxidative stress), the second as an enhancer (here, ABT acting by CYP inhibition). In contrast to the driver, exposure to the enhancer must exceed its effect threshold causing the inhibition of CYP, thus generating synergism. This does not mean that at this dose the enhancer must cause adverse effects such as tissue injury or cell death. If exposure to ABT falls below inhibitory doses, the entire “synergy of evil” collapses and toxicity of OTA will not be increased. However, if doses of OTA are below thresholds that generate reactive oxygen species, combination with an enhancer may—in principle—increase the OTA concentrations in tissues above critical levels where adverse effects occur. From a regulatory perspective, it is therefore important that enhancers stay below doses where the enhancing mechanism becomes active in order to guarantee that a “synergy of evil” does not occur. However, modes of kinetic interaction, such as CYP inhibition, are not yet routinely considered for BMD or NOAEL derivation. The potentially enhancing properties of a substance are currently neglected in mixture risk assessment. The lack of systematic substance screens for their respective inhibitory potential against metabolism or active transport or the overall impact of kinetic interaction at low concentrations remains a data gap that should be addressed in the future.

With regard to the synergism of OTA and ABT, it should be considered that the dose of OTA used in the above example (7.5 mg/kg bw) was chosen in order to illustrate the principle of combined exposure to CYP substrates and inhibitors (Hassan et al. 2022b), although human exposure is known to be much lower. The 95th percentile of exposure of the European population at the upper bound has been reported to be only 0.013 µg/kg bw/day. It appears very unlikely that even a strong enhancer can increase tissue concentrations of the driver by several orders of magnitude.

Human exposure to inhibitors of CYP is possible. For example, various furocoumarins found in grapefruit juice are potent inhibitors of CYP3A and other CYP isoenzymes, which play a central role in the metabolism of many drugs or chemicals (Girennavar et al. 2007; SKLM 2004). The consumption of typical amounts of grapefruit juice may increase the bioavailability/maximum plasma concentrations or the elimination half-life of certain drugs and chemicals and corresponding warnings are thus included in patient information sheets (SKLM 2004). There is consensus that human exposure to plant constituents at doses that cause CYP inhibition should be avoided.

*Toxicodynamic "synergy of evil".* Toxicodynamic “synergy of evil” describes the effect of one substance indirectly enhanced by another, without the substances sharing toxicodynamic mechanisms or interacting kinetically. Although conceptually convincing and well known in pharmacology (both for therapeutic and adverse side effects), it is currently challenging to identify examples, where two or more substances have been demonstrated experimentally to be responsible for a toxicodynamic “synergy of evil” in humans. Acute-on-chronic liver injury appears to fulfil these criteria. Chronic exposure, for example, to alcohol, leads to hepatocytes being more susceptible to acute exposure by certain hepatotoxic drugs. Nonetheless, several aspects of this form of drug induced liver injury still remain insufficiently understood (Zaccherini et al. 2021).

In animal experiments, possible interactions of polychlorinated biphenyls (PCBs) and perchlorate (ClO_4_^−^) have been studied (McLanahan et al. 2007). Both compounds induce hypothyroidism by different mechanisms. The PCB congener used in this study (PCB126) is known to bind to the AhR, inducing uridine diphosphate glucuronosyltransferases (UDPGTs) in hepatocytes and increasing biliary excretion of thyroxine glucuronide, which may lead to hypothyroidism. ClO_4_^−^ inhibits iodide uptake into the thyroid gland, which decreases the production of thyroid hormone. When rats were exposed to relatively high doses of PCB126 prior to ClO_4_^−^ administration, the ClO_4_^−^ dose–response curve was shifted to the right, demonstrating a combined effect. However, the combined effect was less than additive (McLanahan et al. 2007). When rats were exposed to doses of PCB126 and ClO_4_^−^ at or near the NOAEL for each compound, no such combined effect on thyroid indices was observed (McLanahan et al. 2007). The example of the PCB–perchlorate effect illustrates that borderline situations may exist between the toxicokinetic and toxicodynamic “synergy of evil”, since PCB induces the metabolism and excretion of T4, thereby aggravating the effect of perchlorate. However, the canonical toxicokinetic “synergy of evil” as defined in this study requires that the enhancer substance increases the concentration of the driver substance at the target cells of toxicity. In contrast, in the PCB–perchlorate example two different mechanisms converge at the same key event, namely reduced T4 levels, while each substance does not influence the concentration of the other.

A further example observed in mice is the loss of microbiota-mediated negative feedback control on bile acid synthesis in the liver that can be caused by antibiotics. This loss of negative feedback results in increased hepatic bile acid concentrations, which in turn lead to the disruption of bile duct barrier function and, consequently, fatal liver injury. A precondition for fatal liver injury is that other, for example, toxic mechanisms have already caused a cholestatic condition (Schneider et al. 2021). However, it should be considered that large differences between humans and mice exist. Farnesoid X receptor (FXR)-mediated negative feedback mechanisms on bile acid synthesis are more efficient in human than murine hepatocytes.

In another recent study, zebrafish embryos were exposed for 3 days to eight substances selected because they induce craniofacial malformations in zebrafish embryos, either individually or in combination via different mechanisms. Combination of all eight substances at the individual NOAEL caused craniofacial malformations, which showed stronger combined effects compared to the individual substances. However, even though the eight compounds may act upon distinct molecular targets, their adverse mechanisms may still converge at a downstream target to induce craniofacial malformation (van Der Ven et al. 2022). To our knowledge, complex mixtures representative of realistic combined human exposure have not been experimentally studied.

Due to the limited availability of adequate in vivo studies, it remains debatable, whether substances acting by mechanisms converging in a shared key event, such as mitochondrial toxicity, oxidative stress or accumulation of intracellular bile acid concentrations, should be regarded under the concept of the toxicodynamic “synergy of evil” or the “multi-headed dragon”. Future research may answer the question, whether it is more adequate to limit the “multi-headed dragon” concept to substances that act exactly by the same mechanism or to open it to substances that converge at specific key events. As the EFSA CAG concept suggests, there may either be a certain overlap of the concepts, or we do not yet sufficiently understand how compounds affecting different nodes of a complex network act together towards a common adverse effect (Goodson III et al. 2015; Miller et al. 2017).

### Hypothesis of the “revolting dwarfs”

**Fig. 4:**
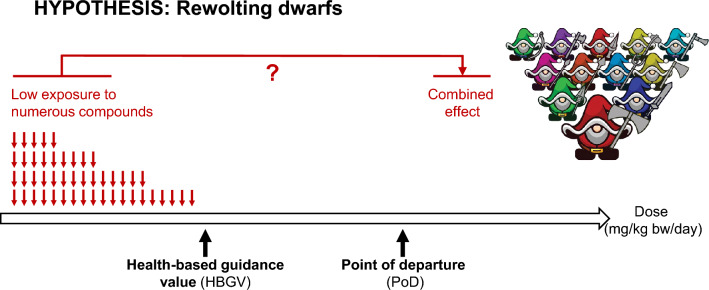
Concept of the revolting dwarfs

There is no doubt that humans are continuously exposed to complex mixtures, *i.e.* very low doses of numerous substances at levels below the HBGV in food or via environmental as well as occupational exposure (Escher et al. 2020). For many of these substances, only trace amounts are detectable in the human body. Here, we define a “dwarf” as a substance present in a mixture below its individual HBGV so that human exposure is below the PoD by more than the OAF. Individually, none of these low-dose substances causes adverse effects (Fig. 4). According to the “revolting dwarfs” hypothesis, these substances may nevertheless cause adverse effects in humans due to sub-additive, additive or synergistic activities despite their low doses, simply because of their sheer number. Although not under the name of “revolting dwarfs”, the basic idea has already been around for some time in the field of toxicology, albeit with some note of caution as all these substances would indeed require to be present at their targets simultaneously or in close succession. This requirement of concurrent exposure at a target site puts limits to the concept’s applicability, particularly in terrestrial organisms. The concept was recently put forward again, e.g. by Baumer et al. (2021) and Escher et al. (2021). Accordingly, it has been hypothesised by the authors that “individual contaminants may be present at very low concentrations, far below any concentration expected to cause adverse effects on their own but acting together in mixtures their biological activity may lead to detectable effects" (Escher et al. 2021).

Combining such “dwarfs” in a designed mixture experiment to explore whether an additive effect is observed is pragmatic and in principle easily applicable. However, it is necessary to note that using this approach to study mixtures experimentally has a significant limitation if it assumes that the relative potency ratios between the individual “dwarfs” at their PoD are the same as those at their respective PoD/OAF values. In other words, it linearly extrapolates from the PoD to the HBGV, where in reality the respective part of the dose–response relationship may not be linear at all and the dose causing an effect at the HBGV may in fact be higher or lower than the HBGV.

The question nevertheless remains if these dwarfs, at such individually harmless exposure levels may act together to cause adverse effects. The “revolting dwarfs” hypothesis has been addressed by several animal experiments. In a historical landmark study, Ito and colleagues exposed rats to 20 pesticides (19 organophosphorus and one organochlorine substance) via their diet. Each pesticide was used at its individual ADI (Ito et al. 1995). The authors studied the formation of pre-neoplastic lesions in the liver initiated by N-nitrosodiethylamine in an 8-week exposure model. This endpoint was relevant since liver carcinogenicity was reported or suspected for high doses of some of the tested substances, such as malathion or methidathion (Ito et al. 1995). Importantly, the mixture of these 20 pesticides at their ADI did not enhance pre-neoplastic lesions. In a second experiment, 40 pesticides representing examples of high-production volume chemicals, or 20 pesticides with suspected carcinogenicity, were added to the diet at the respective ADI levels and tested by a multi-organ 28-week carcinogenicity protocol (Ito et al. 1995). The mixtures did not induce carcinogenicity in any organ. Therefore, the 40 substances at individually harmless doses did not act together to cause adverse effects for the endpoint analysed in rats. For theoretical considerations, it would be interesting to combine even more substances at similarly low doses to learn how many substances are required until an adverse mixture effect can be observed. On the other hand, it is extremely unlikely that humans are simultaneously exposed to 40 pesticides at the doses used in rats in the study of Ito et al. (1995).

Numerous additional studies addressed the relevance of co-exposures using different mixtures including in vivo readouts (*e.g.* Elcombe et al. 2022; Heise et al. 2018; Houtman et al. 2014; Ma et al. 2023; Perez-Carreon et al. 2009; Rieke et al. 2017; Sams and Jones 2011; Schmidt et al. 2016). Some studies used combinations of substances at the NOAEL of their respective assays. An important question is which criteria should be fulfilled by studies on mixtures in the future. Studies are required that test mixtures that are representative of what humans are actually exposed to. One possible approach is to identify human exposure to “dwarf” compounds and test these mixtures at current exposure levels and at proportionally increased levels in experimental animals. An alternative approach is to define the individual doses in a mixture based on the PoD (testing combinations of for example 1/3, 1/10, 1/30, 1/100 of the PoD), to learn if the combination effects are sub-additive, additive or higher than additive in an experimental animal. In this type of experiment, it will be important to also generate reliable data of the individual substances (besides the mixtures) using the same exposure conditions and readouts to guarantee comparability between the individual substances and mixtures. Moreover, species differences need to be considered between humans and test animals. The conduction of similar experiments in vitro should account for relevant kinetic processes, *e.g.* by conducting PBTK modelling (Ma et al. 2023). Alternatively, testing complex “real-life” mixtures can be done using a whole-mixture-based in vitro strategy, as currently explored within the Green Deal project PANORAMIX (Escher et al. 2022).

## Grouping by “cumulative assessment groups” (CAGs)

As discussed under the concept of the “multi-headed dragon”, substances acting by the same mechanism in a common target cell may cause additive effects. The concept of CAGs goes further in that it postulates additive effects, if substances belong to a common “cumulative assessment group” (CAG), whereby CAGs often refer to common target organ pathologies (Boberg et al. 2021; EFSA Scientific Committee et al. 2019; EFSA Scientific Committee et al. 2021; van den Brand et al. 2022; Wohlleben et al. 2023). The concept may be legitimate for a conservative first tier assessment, e.g. for prioritisation purposes. However, considering a common organ to evaluate the hazard of a chemical mixture without further refinement is too generic to assume additive effects realistically. There are several examples showing that this concept needs further refinement to the level of target molecules or target pathways. Organ toxicity, for instance, hepatotoxicity, may be caused by numerous mechanisms, such as activation of several nuclear receptors (Knebel et al. 2018), alteration of lipid metabolism (Brecklinghaus et al. 2022a), mitochondrial toxicity (Ghallab et al. 2019; Pessayre et al. 1999), protein or lipid binding (Agarwal et al. 2012; Sezgin et al. 2018), and inhibition of carriers of hepatocytes or cholangiocytes (Brecklinghaus et al. 2022b; Ghallab et al. 2022; Remetic et al. 2022), among others. Due to the multitude of possible mechanisms, grouping for additivity by target organ thus always needs to include mechanistic considerations.

Also grouping principles for additive effects based on specific pathologies, such as cholestasis (Boberg et al. 2021; Foster et al. 2020) may be too rough. Although more specific than simply grouping according to the target organ, and therefore representing a legitimate, slightly less conservative second tier assessment, grouping according to pathology, likewise, is too unspecific. As to the example of cholestasis, it should be considered that the biliary tract is complex, and different mechanisms may be active in the canalicular network, interlobular ducts and large ducts (Jansen et al. 2017; Vartak et al. 2021b). Numerous carriers orchestrate the uptake of bile acids and further organic solutes from the sinusoidal blood into hepatocytes. In contrast, other groups of carriers are responsible for secretion from hepatocytes into bile canaliculi (Vartak et al. 2021a). Several mechanisms control the secretion of water by cholangiocytes, including cAMP-, PKA- and IP3-dependent mechanisms (Vartak et al. 2021a). Moreover, inflammatory processes may cause bile duct obstructions, while others compromise hepatocyte polarity, and consequently the efficacy of bile drainage via bile canaliculi (Vartak et al. 2016). This case serves as an example to illustrate that even a common pathology, such as cholestasis, may remain too vague to define substances that will act additively. Rather, additive effects may, for example, occur when the same carrier is inhibited by two or more substances. A well-studied example is the inhibition of the canalicular bile salt secretion carrier BSEP (Brecklinghaus et al. 2022b). When a substance inhibits BSEP, the secretion of bile acids from hepatocytes into bile canaliculi is reduced, which may lead to an increase in hepatocellular bile acid concentrations above cytotoxic thresholds. It is plausible that several substances that inhibit BSEP will act additively. Consistent with the latest grouping guidance of EFSA, these considerations demonstrate that common molecular mechanisms are the most reliable grouping principle for possible additive effects, as similarly discussed for nuclear receptors in the previous section (EFSA Scientific Committee et al. 2021).

## Grouping into common kinetic groups (CKGs)

Another limitation of classical grouping is the disregard for kinetic interactions. As explained above for OTA and ATB, disrupting the metabolic clearance of a toxic substance may increase its concentration in target tissues and consequently its adverse effects. In addition, the inhibition of active efflux or the induction of active uptake transporters may also affect the toxicity of a substance (Braeuning et al. 2022). In substance-based risk assessment and threshold derivation, such modes of interaction are not considered and remain a data gap in mixture toxicity. Consequently, Braeuning et al. (2022) proposed to group substances based on their ability to induce or inhibit metabolic enzymes and active transporters. In addition, more research is required to understand the impact of solvents and surfactants on the passive transport of toxic substances to their target sites (Karaca et al. 2021).

## Conclusions and recommendations

Based on the currently available scientific evidence, the present study did not find convincing proof that human health is at risk due to the combined exposure to many substances that are below their individual HBGV at current exposure conditions, arguing against the hypothesis of the “revolting dwarfs”. A “synergy of evil” may in principle take place, if there is actually an “enhancer substance” at sufficiently high concentrations in target tissues that co-occurs with a “driver substance”. However, if sufficient information would be available, this could be prevented by the adequate risk management of the enhancer and the driver as individual substances. Substances acting according to the “multi-headed dragons” principle by affecting the same molecular mechanism in the same target cells may act additively, as reported for dioxin-like substances. This observation highlights the importance of elucidating the molecular mechanisms leading to adverse outcomes, as well as of studying the compounds’ toxicokinetics.

Based on the abovementioned data and described principles of mixture toxicity, we conclude that the available evidence does neither support the requirement of additional generic safety factors nor of generic MAFs. Despite the need for a more systematic review and screenings for substances potentially causing mixture effects, the weight of evidence indicates that mixture effects of concern for human health are likely rare when human exposure is below the HBGV of the individual substances. Rather, substances with relatively low ratios between PoD and currently occurring exposure require a targeted risk evaluation; this is even more critical when these substances share common molecular mechanisms or interact kinetically. Therefore, to protect the general population as well as highly exposed subpopulations we propose that resources should be directed towards risk evaluation of substances with a relatively low margin of exposure (MoE, ratio of the POD to the predicted, or estimated human exposure level) or RCR.

A recommendation for future research is that the overview of substances to which human populations are exposed should be improved. This should lead to a comprehensive overview of all substances from different sources, such as food, environment and workplace, which are often regulated by different expert panels. Such overviews should include information on the exposure of human populations, HBGV and PoDs, molecular mechanisms responsible for the toxicity and toxicokinetics including information on the affected cell types. Often, a major hurdle is the lack of reliable exposure information for such chemicals based on the REACH standard information requirements, which prevents reliable RCR or MoE calculations and requires the use of worst-case assumptions that may lead to a significant overestimation of the risk. More accurate use and exposure information should therefore be provided on a mandatory basis.

New approach methodologies (NAMs) will be helpful in screening and prioritising substances of concern. In particular, toxicokinetic grouping into CAGs combined with co-exposure assessment will be a useful tool for the identification of substances with potentially synergistic effects. In addition, there should be a comprehensive compilation of all current knowledge on the target organs/target cell types and molecular mechanisms of toxicity. If substances share the same target organ and act, for example, via the same nuclear receptor or by inhibition of the same carrier, additive effects appear to be probable. In this case, the TEF concept, which is currently successfully applied for dioxin-like substances, could be implemented, even if the substances do not belong to the same substance class. Thus, it may be applied to substances acting by the same molecular mechanisms and on the same target cells.

In conclusion, considering the available scientific evidence there are hardly any supporting arguments for generic mixture assessment/allocation factors, neither by the available conceptual data nor by in vivo experiments. This is not to say that mixture toxicity will not pose a risk for particular substances and exposure scenarios, particularly in the presence of drivers. However, in light of the aforementioned concepts it seems questionable that these are best approached generically. Rather, more specific data-driven measures are warranted to improve the evaluation of substances with a low margin of exposure or a low risk characterisation ratio.
